# Modular Organization of Engulfment Receptors and Proximal Signaling Networks: Avenues to Reprogram Phagocytosis

**DOI:** 10.3389/fimmu.2021.661974

**Published:** 2021-04-19

**Authors:** Emily A. Britt, Vanessa Gitau, Amara Saha, Adam P. Williamson

**Affiliations:** Department of Biology, Bryn Mawr College, Bryn Mawr, PA, United States

**Keywords:** phagocytosis, engulfment, immune receptor, signal transduction, macrophage reprogramming, chimeric antigen receptor, immunotherapy

## Abstract

Transmembrane protein engulfment receptors expressed on the surface of phagocytes engage ligands on apoptotic cells and debris to initiate a sequence of events culminating in material internalization and immunologically beneficial outcomes. Engulfment receptors are modular, comprised of functionally independent extracellular ligation domains and cytosolic signaling motifs. Cognate kinases, adaptors, and phosphatases regulate engulfment by controlling the degree of receptor activation in phagocyte plasma membranes, thus acting as receptor-proximal signaling modules. Here, we review recent efforts to reprogram phagocytes using modular synthetic receptors composed of antibody-based extracellular domains fused to engulfment receptor signaling domains. To aid the development of new phagocyte reprogramming methods, we then define the kinases, adaptors, and phosphatases that regulate a conserved family of engulfment receptors. Finally, we discuss current challenges and opportunities for the field.

## Introduction

**Figure 1:**
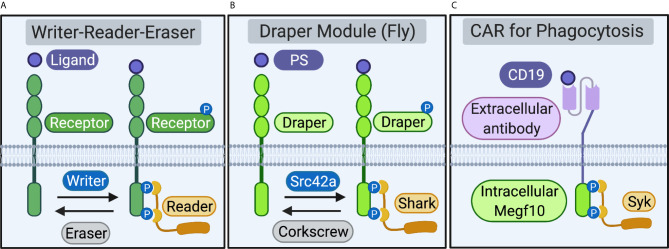
Receptors and proximal writer-reader-eraser modules. **(A)** Schematic of a generalized receptor and writer-reader-eraser module. Receptor ligation induces phosphorylation of a receptor intracellular signaling domain by a writer kinase. The phosphorylated receptor recruits a reader adaptor protein. An eraser phosphatase removes phosphorylation marks on the receptor intracellular domain, returning the system to its resting state. **(B)** A writer-reader-eraser module regulates signal transduction through the Draper receptor. Draper, expressed on fruit fly phagocytes, recognizes phosphatidylserine (PS) or other ligands. Phosphorylation of the Draper intracellular domain by the kinase Src42a induces recruitment of the adaptor kinase Shark. The phosphatase Corkscrew erases phosphorylation marks on the intracellular domain of Draper. **(C)** Chimeric Antigen Receptor for Phagocytosis (CAR-P). Synthetic CAR-Ps bind cancer antigens (e.g. the B cell antigen CD19) *via* an extracellular single-chain antibody fragment (scFv). Extracellular antibody ligation induces phosphorylation of engulfment receptor signaling domains (e.g. the intracellular domain of mouse Megf10). Phosphorylation of the Megf10 receptor signaling domain on CAR-P recruits the Syk adaptor, a cognate reader for murine Megf10. Schematic created in BioRender (biorender.com).

The Megf10/Draper/CED-1 receptors, an ancient family of single-pass transmembrane proteins, enable phagocytes across phyla to initiate the internalization of apoptotic cells, synapses, and cell debris (1–3). Ligation of engulfment receptor extracellular domains to “eat me” signals on the target, including the complement protein C1q and the lipid phosphatidylserine (PS), induces receptor phosphorylation by “writer” kinases. Phosphorylation recruits “reader” proteins that initiate a series of cytoskeletal and membrane-remodeling events that enable the phagocytes to ingest large targets and digest internalized material in intracellular compartments. “Eraser” phosphatases then dephosphorylate engulfment receptors to return the receptor to its unphosphorylated resting state (4). Writer-reader-eraser modules that regulate receptor activation are common components of vertebrate immune cell signaling pathways (**Figure 1A**). Notable examples include the Src-Syk-CD45 writer-reader-eraser network that controls Fc-receptor signaling and the Lck-ZAP70-CD45 module that tunes activation the T cell receptor (TCR) (5).

The Megf10/Draper/CED-1 receptors are composed of functionally separable extracellular ligation and intracellular signaling modules. The modularity of engulfment receptors enables phagocyte reprogramming, as replacement of the extracellular domain with a new recognition element directs phagocytes to identify and ingest new targets (6–8). The organization of engulfment signaling is also modular at the level of the receptor proximal signaling networks. Engulfment receptors function in concert with effector modules comprised of kinases, adaptors, and phosphatases that initiate the engulfment signaling program. Here, we review recent work that highlights the promise of reprogramming phagocytosis to understand basic immunology in pre-clinical settings. We then use the modular structure of synthetic engulfment receptors as an inlet to define and compare the writers, readers, and erasers that regulate initiation of the engulfment signaling program through the Megf10/Draper/CED-1 receptors (**Figure 1B**).

## Reprogramming Phagocytosis Using Chimeric Antigen Receptors

In multiple immune cell types, including T cells, phagocytes, and natural killer cells, replacement of an extracellular domain with a single-chain antibody fragment (scFv) can reprogram an immune cell towards the cognate antigen of a specific scFv (7, 9, 10). Reprogramming immune cell signaling has ushered in a new suite of methods to engineer the immune response to target cancer (11). Analogous to Chimeric Antigen Receptors in T cells (CAR-T), the modular organization of engulfment receptors enables investigators to reprogram phagocytes to identify and eliminate targets of therapeutic interest.

The modular organization of the T cell, Fc-, and phagocyte receptors enables the reprogramming of immune cells to recognize and respond to non-native targets of therapeutic interest, including cancer antigens (6, 7, 9). Multiple groups have successfully reprogrammed macrophages to recognize cancer antigens by introducing synthetic Chimeric Antigen Receptors for Phagocytosis (CAR-Ps) (6, 7). Fusing the Megf10 or Fc-receptor signaling domains to an scFv recognizing the B-lymphocyte antigen CD19 programmed mouse macrophages to ingest antigen-coated beads and cancer cells (**Figure 1C**) (7). Surprisingly, chimeric receptors expressed in macrophages composed of extracellular scFvs fused to the T cell receptor signaling domain CD3ζ also drive engulfment (6, 7). Importantly, Klichinsky et al. also demonstrated the following: anti-cancer activity in humanized mouse models; induction of a pro-inflammatory tumor microenvironment; and antigen cross presentation to boost anti-tumor T cell responses (6). Collectively, coupling scFv-based extracellular modules to ITAM-based intracellular signaling domains from Megf10, Fc-receptor, and the TCR, presents an attractive strategy to reprogram macrophages towards therapeutically relevant targets linked to both hematopoietic malignancies and solid tumors (6, 7).

At present, methods primarily rely on extracellular antibody-based modules that recognize antigens with high affinity. However, high-affinity CAR-Ps may signal in a manner that does not reflect endogenous receptor mechanisms. Recent work demonstrates that T cells transduce signals through CAR-T independently of a key T cell signaling protein required by physiological T cells called Linker of Activated T cells (LAT) (12). Future work for the field will require engineering molecules that more closely reflect the biology of native engulfment receptors, perhaps by developing new CARs that combine multiple signaling modules to orient cellular responses towards desired immunological outcomes. To learn how engulfment receptors interface with proximal signaling modules, we systematically defined the writer, reader, and erasers that regulate the Megf10/Draper/CED-1 family.

## Modular Organization of Engulfment Signaling Networks Across Phyla

Application of the writer-reader-eraser framework enables conserved rules and regulatory principles underlying engulfment to emerge from this complex collection of receptors and their proximal signaling networks. Here, writers refer to protein tyrosine kinases that create reversible binding sites for readers. The readers, SH2- and PTB-domain-containing proteins, initiate the engulfment program by activating “downstream” signaling modules that remodel the actin cytoskeleton and cell membranes (13). The cycle is completed by erasers, protein tyrosine phosphatases that return the system to its deactivated state.

### Megf10: Clearing Corpses and Shaping Synapses in the Mouse Brain

Megf10, a receptor expressed on phagocytic astrocytes in the mouse brain, promotes synapse elimination (14) and apoptotic cell clearance (1). Megf10 also plays roles in retinal patterning (15) and elimination of amyloid-β (16). After recognition of its ligand, the complement protein C1q (1), Src-family kinase writers phosphorylate Tyrosine (Tyr) residues within the Megf10 Immunoreceptor Tyrosine-based Activation Motif (ITAM) (17). In addition to its ITAM, the cytosolic tail of Megf10 encodes an NPxY motif (18), a domain capable of recruiting cytosolic signaling proteins *via* direct interaction with phosphotyrosine binding (PTB) domains. Consistent with a writer function for Src-family kinases, treating HeLa cells that overexpress Megf10 with the Src-family kinase inhibitor PP2 decreases engulfment of microspheres by HeLa cells transfected with Megf10 (17). Thus, multiple Src-family kinases can create binding sites for readers on the cytosolic tail of Megf10.

The tandem SH2-domain-containing kinase Syk is a Megf10 reader that interacts with phosphorylated ITAM residues on the cytosolic domain of Megf10 (17). Though the specific actions of Syk that enable phagocytosis in glia are unclear, Syk is an important engulfment effector in macrophages and plays a role in remodeling the actin cytoskeleton when bound to the Fc-receptor (19). The PTB-containing protein GULP is upregulated in reactive astrocytes after transient ischemic injury, an expression pattern similar to Megf10 (20). Thus, in addition to Syk, GULP is a candidate to interact with the intracellular domain of Megf10 *via* an NPxY motif.

A specific eraser for Megf10 is not reported, though mouse astrocytes express SHP-2 (PTPN11) mRNA (21). SHP-2 is a cytosolic protein tyrosine phosphatase that dephosphorylates the ITAM-bearing C-type lectin receptor that governs anti-fungal immunity (22). In sum, Megf10 is an invariant single-pass transmembrane engulfment receptor. A module comprised of multiple Src-family kinases and Syk enables Megf10 to initiate the clearance of apoptotic cells and synapses.

### Draper and a Proximal Writer-Reader-Eraser Module Initiate Engulfment by Fly Phagocytes

Phagocytes in the fruit fly *Drosophila melanogaster* clear apoptotic corpses and axonal debris to maintain tissue homeostasis and repair the nervous system after injury. Engulfment in flies is carried out by an array of cell types including hemocytes (23), epithelial cells (24), and glia (2, 25). The Draper receptor, expressed in each of these cell types, is comprised of an extracellular emlin (EMI) domain that facilitates protein-protein interactions (26) and nimrod (NIM) repeats, EGF-like domains found on multiple fruit fly innate immune receptors (27). The intracellular signaling domain of Draper contains two phosphotyrosine-based signaling sequences that serve as candidate sites for phosphorylation by Src-family kinase writers: an NPxY and an ITAM.

Ligation of Draper to phosphatidylserine induces phosphorylation of the cytosolic signaling domain of Draper (8, 28). Intriguingly, Draper recognizes a diverse array of additional ligands in other contexts including the proteins Pretaporter (29) and DmCaBP1 (30) and the bacterial surface molecule Lipoteichoic acid (31). Once ligand-receptor binding occurs, clustered Draper is rapidly phosphorylated in phagocyte plasma membranes (8). At least 5 residues on the cytoplasmic signaling domain of Draper, including at least 4 outside the ITAM, are phosphorylated by the kinase Src42a (8, 32). The kinase Src42a is the writer in this system, creating a binding site for the tandem SH2-domain kinase Shark (32) (**Figure 1B**).

The cytosolic protein tyrosine phosphatase Corkscrew, which binds an alternatively spliced isoform of Draper called Draper-II *via* an Immunoreceptor Tyrosine-based Inhibitory Motif (ITIM), is the eraser for Draper (33). Corkscrew is capable of dephosphorylating Draper and, intriguingly, loss of Corkscrew *in vivo* inhibits the ability of glia to respond to secondary injury (33). The interdependence of the writer, reader, and eraser in the fly system underscores the importance of returning this immune cell signaling system to its ground state to respond to future insults.

Collectively, a wealth of genetic, biochemical, and cell biological evidence supports the model that engulfment through Draper is carried out by a writer-reader-eraser module comprised of a writer kinase Src42a, an SH2-domain-containing reader Shark, and an eraser Corkscrew. As writers, readers, and erasers remain to be determined in other organisms, the fruit fly represents an exceptional model system for defining the mechanisms underlying engulfment through the Megf10/Draper/CED-1 receptors.

### CED-1 in the Worm: A Divergent Engulfment Receptor

Genetic work in the nematode *Caenorhabditis elegans*, including the discovery of the CED-1 receptor (3), founded the engulfment field. *C. elegans* phagocytes clear dead and dying cells using two pathways governed by engulfment factors expressed by ced genes. One pathway includes ced-2, ced-5, and ced-12 while the other pathway includes ced-1, ced-6, and ced-7 (34). These pathways converge on actin to facilitate rearrangements that enable engulfment (35).

Corpse clearance by phagocytic cells in the worm is initiated when cells neighboring the apoptotic cell release the secretory protein Transthyretin-related 52 (TTR-52) (36). TTR-52 binds PS on the corpse and serves as a bridging molecule to the receptor CED-1. CED-1 ligation to TTR-52 induces activation of the CED-6 and CED-7 signaling pathway. Like Draper and Megf10, the extracellular domain of CED-1 contains EMI and EGF-like domains (3, 26). The cytosolic signaling tail on CED-1 contains a possible Tyrosine phosphorylation site on a single YxxL motif (3). The tail also contains an NPxY motif with a Tyr residue. However, *in vitro* evidence indicates that NPxY motifs may bind PTB-domain-containing proteins such as CED-6 in the absence of Tyr phosphorylation (37). Thus, our application of the writer-reader-eraser framework to the *C. elegans* CED-1/CED-6/CED-7 engulfment module reveals that while worms do encode candidate writer kinases and eraser phosphatases, the CED-1 receptor may perform engulfment in the absence of Tyr phosphorylation.

## Conclusions, Current Challenges, and New Opportunities

### Comparison of Writer-Reader-Eraser Modules Used by Phagocytes Across Phyla

Our application of the writer-reader-eraser framework to define the proximal signaling networks that regulate the Megf10/Draper/CED-1 receptor family revealed broad mechanistic similarities and intriguing differences (**Table 1**). All three receptors contain at least one cytoplasmic Tyr residue in an ITAM or NPxY motif, potential phosphorylation sites for kinase writers. At least one reader protein interacts with Megf10, Draper, or CED-1 to initiate the complex series of cytoskeletal and membrane-remodeling events that power material internalization. Megf10 and Draper recruit a Syk-family kinase reader and CED-6/GULP adaptor proteins. CED-1 binds the reader CED-6. Collectively, the proximal writers and readers governing Megf10 and Draper function are highly similar to one another. Because the CED-6 adaptor protein may bind CED-1 independently of Tyr phosphorylation, CED-1 may function *via* a distinct activation mechanism that does not require a writer kinase. Less is known about the eraser phosphatases that negatively regulate the Megf10/Draper/CED-1 receptors. The eraser for Draper, a SHP-2 tyrosine phosphatase ortholog called Corkscrew, works by binding an inhibitory splice isoform of Draper (33). The cognate phosphatases for Megf10 and CED-1, though presumably expressed by the relevant phagocytes, remain unreported.

**Table 1:** Summary of receptors and writer-reader-eraser modules used across biological processes and organisms.

Biological Process (Organism)	Receptor	Ligand(s)	Writer	Reader	Eraser	References
Phagocytosis of opsonized targets (Mouse)	Fc-receptor	Antibody fragment crystallizable (Fc) region	Src-family kinases	Syk	CD45	(38, 39)
T-cell receptor activation (Mouse)	T-cell receptor	Peptide-MHC	Lck	ZAP70	CD45	(40)
Targeting hematologic malignancies (Mouse)	Antibody-based Chimeric Receptors	CD19, CD22	Not reported	Syk	Not reported	(6, 7)
Targeting solid tumors (Mouse)	Antibody-based Chimeric Receptors	Mesothelin, HER2	Not reported	Syk	Not reported	(6)
Engulfment of apoptotic cells and synapses (Mouse)	Megf10	C1q	Src-family kinases	Syk	Not reported	(1, 17)
Engulfment of apoptotic cells and axonal debris (Fly)	Draper	Phosphatidylserine, Lipoteichoic acid, Pretaporter, DmCaBP1	Src42a	Shark	Corkscrew	(8, 28–33)
Engulfment of apoptotic cells (Worm)	CED-1	TTR-52	Not reported	CED-6	Not reported	(3, 35, 36)

Megf10, Draper, and CED-1 differ remarkably in their ligand specificities, perhaps a result of their evolutionary divergence and the large number of different “eat me” signals presented on the surface of apoptotic cells and other targets (41). Megf10 binds the complement protein C1q to initiate the engulfment of apoptotic cells (1). Draper initiates engulfment by interacting with diverse ligands including the lipid PS and the proteins DmCaBP1 and Pretaporter (8, 28–30). Finally, CED-1 recognizes the secreted protein TTR-52 that serves as A bridge between CED-1 and PS (36). Perhaps the diversity of ligands that the Megf10/Draper/CED-1 receptors recognize provides the mechanistic basis for reprogramming the receptors using synthetic extracellular domains. We hope that further study of engulfment receptors and their proximal signaling modules will enable researchers to design a new generation of synthetic signaling systems comprised of CARs that interact with engineered intracellular signaling modules.

### Reprogramming Phagocytes Toward New Targets

In theory, new CAR-Ps that enable phagocytes to bind any extracellular cell surface protein or secreted molecule should program phagocytes to ingest non-native targets. At present, however, implementing CAR-Ps in a clinical context requires laborious editing protocols to manipulate autologous cells. Thus, the use of CAR-Ps as therapies to treat human disease remains a goal for the future. For now, CAR-Ps are emerging as important tools to define basic immunology by elucidating the mechanisms used by receptors expressed across diverse phagocyte populations.

As investigators continue to design and express new receptors, it will be critical to move beyond internalization to learn how engulfed material is targeted to promote specific immunological outcomes. For example, recent work linking the receptor DNGR-1 to antigen cross-presentation suggests that expression of a DNGR-1-based CAR-P in Dendritic Cells may drive the efficient cross-presentation of cancer antigens (42). In the long term, replacing cell-based CAR-Ps with bi- or tri-specific antibodies that couple desired phagocyte populations exposing known cell surface molecules to disease-associated antigens may prove to be viable, inexpensive strategies that could democratize access to new therapies.

### Reprogrammed Phagocytes That Facilitate Nondestructive Immune Responses

In conclusion, further study of engineered phagocytes could spur the development of therapeutic interventions that cover a broad array of “accommodation” immune archetypes: active, nondestructive responses that, among other outcomes, promote wound healing and tissue repair (43). Phagocytes are particularly well suited to promote nondestructive responses because their endogenous functions include clearing apoptotic cells without inducing local inflammatory responses. In this review, we focused on modular receptor signaling systems that offer opportunities to reprogram phagocytes to ingest cancer cells. A key future challenge for the field is to develop phagocyte engineering strategies targeted at a broader range of diseases and cell populations. We anticipate that the development of new chimeric receptors that connect extracellular ligation to synthetic intracellular signaling modules will lead to exciting discoveries at the frontier of this nascent, expanding field.

## Author Contributions

EB, VG, and AS contributed equally to this work and are listed alphabetically. EB completed an analysis of the Megf10 primary literature and drafted and edited the article. VG completed an analysis of the CED-1 primary literature and drafted and edited the article. AS completed an analysis of the Draper primary literature and drafted and edited the article. AW drafted and edited the article. All authors contributed to the article and approved the submitted version.

## Funding

EB, VG, AS – Undergraduate Summer Science Research Funding, Bryn Mawr College. AW – Research Startup Funds, Bryn Mawr College.

## Conflict of Interest

AW is an inventor on U.S. patent application WO2020097193 “Chimeric antigen receptors for phagocytosis” and receives licensing fees for the work described in this article.

The remaining authors declare that the research was conducted in the absence of any commercial or financial relationships that could be construed as a potential conflict of interest.
